# Assessment and Improvement of Drug Data Structuredness From Electronic Health Records: Algorithm Development and Validation

**DOI:** 10.2196/40312

**Published:** 2023-01-25

**Authors:** Ines Reinecke, Joscha Siebel, Saskia Fuhrmann, Andreas Fischer, Martin Sedlmayr, Jens Weidner, Franziska Bathelt

**Affiliations:** 1 Institute for Medical Informatics and Biometry Carl Gustav Carus Faculty of Medicine Technische Universität Dresden Dresden Germany; 2 Center for Evidence-Based Healthcare, Carl Gustav Carus Faculty of Medicine, Technische Universität Dresden Dresden Germany; 3 Hospital Pharmacy, University Hospital Carl Gustav Carus Dresden Germany

**Keywords:** secondary usage, Observational Medical Outcomes Partnership, OMOP, drug data, data quality, Anatomical Therapeutic Chemical, ATC, RxNorm, interoperability

## Abstract

**Background:**

Digitization offers a multitude of opportunities to gain insights into current diagnostics and therapies from retrospective data. In this context, real-world data and their accessibility are of increasing importance to support unbiased and reliable research on big data. However, routinely collected data are not readily usable for research owing to the unstructured nature of health care systems and a lack of interoperability between these systems. This challenge is evident in drug data.

**Objective:**

This study aimed to present an approach that identifies and increases the structuredness of drug data while ensuring standardization according to Anatomical Therapeutic Chemical (ATC) classification.

**Methods:**

Our approach was based on available drug prescriptions and a drug catalog and consisted of 4 steps. First, we performed an initial analysis of the structuredness of local drug data to define a point of comparison for the effectiveness of the overall approach. Second, we applied 3 algorithms to unstructured data that translated text into ATC codes based on string comparisons in terms of ingredients and product names and performed similarity comparisons based on Levenshtein distance. Third, we validated the results of the 3 algorithms with expert knowledge based on the 1000 most frequently used prescription texts. Fourth, we performed a final validation to determine the increased degree of structuredness.

**Results:**

Initially, 47.73% (n=843,980) of 1,768,153 drug prescriptions were classified as structured. With the application of the 3 algorithms, we were able to increase the degree of structuredness to 85.18% (n=1,506,059) based on the 1000 most frequent medication prescriptions. In this regard, the combination of algorithms 1, 2, and 3 resulted in a correctness level of 100% (with 57,264 ATC codes identified), algorithms 1 and 3 resulted in 99.6% (with 152,404 codes identified), and algorithms 1 and 2 resulted in 95.9% (with 39,472 codes identified).

**Conclusions:**

As shown in the first analysis steps of our approach, the availability of a product catalog to select during the documentation process is not sufficient to generate structured data. Our 4-step approach reduces the problems and reliably increases the structuredness automatically. Similarity matching shows promising results, particularly for entries with no connection to a product catalog. However, further enhancement of the correctness of such a similarity matching algorithm needs to be investigated in future work.

## Introduction

### Background

Over the last decade, the amount of electronically available data in the health care domain has increased enormously worldwide. Much of the data is generated during the processing of administrative claims, through documentation processes in electronic health records (EHRs) performed during patient treatments, or via data feeds from mobile devices providing patient-reported outcomes. Therefore, it is not surprising that real-world data (RWD) are becoming more important for health care research. RWD studies can be considered complementary to randomized controlled trials (RCTs), as they allow the results of RCTs to be confirmed in much larger cohorts and over a longer period. Compared with RCTs, RWD studies allow for better external validity and better generalizability, and they not only offer opportunities for long-term surveillance of drug products but also are cost-effective and time-saving [1].

Drug surveillance systems, such as the US Food and Drug Administration’s Sentinel initiative, are critical for promoting postmarket drug safety [2-8]. The European Medicines Agency has also started to establish research infrastructure based on RWD to support pharmacovigilance [9]. In addition, the European Health Data and Evidence Network [10] emerged to establish transnational research networks based on a common data model that enables standardized RWD and methods for observational studies to generate real-world evidence. Recently, the European Health Data and Evidence Network has begun to collaborate with the European Medicines Agency to address COVID-19 [11].

However, the original purpose of RWD generation during patient treatment is not primarily aimed at its use in research. Therefore, notable problems have been identified regarding the replication and validity of observational research results based on RWD. To ensure the reliability and robustness of the results from RWD research, these issues have to be addressed, as they become even more important when observational studies are conducted across countries at large scale.

Data harmonization, the use of international standards and terminologies, a common data model, methods, and tools for data analyses that increase the reproducibility of results are needed [12]. These gaps are being addressed by the International Observational Health Data Sciences and Informatics community, which provides the common data model called the Observational Medical Outcomes Partnership (OMOP) and standardized analysis tools based on OMOP. It also includes standardized vocabularies that contain translations between national terminologies and internationally acknowledged terminologies, for example, Systematized Nomenclature of Medicine-Clinical Terms, Logical Observation Identifiers Names and Codes, Anatomical Therapeutic Chemical (ATC) classification, and RxNorm [13]. OMOP allows RWD to be stored in the same way, regardless of data origin, thus ensuring the use of RWD in international, large-scale observational studies. Compared with similar projects such as Informatics for Integration Biology and the Bedside or the National Patient-Centered Clinical Research Network, Observational Health Data Sciences and Informatics-OMOP meets the needs of observational RWD studies well [14]. Many RWD studies on OMOP have shown that drug data at the ingredient level are sufficient to answer their research questions [15]. Although drug data with details on dosage and units for drug exposure can be important for observational research on drug effectiveness and drug safety with the same drug at different doses, the availability of the drug ingredient is the least common denominator and the basic requirement for drug-related RWD studies on OMOP. Therefore, drug prescription data must be available in a structured format that does not necessarily include the name of the drug product but at least the ingredient information. For drug utilization research, the World Health Organization recommends the use of ATC classification, which divides drugs into different groups based on the organ or system on which they act [16]. ATC classification includes a hierarchy based on 5 different levels, with ATC level 5 being the chemical substance that represents the active ingredient of a drug product [17]. Each approved drug product on the market is assigned a specific ATC level 5 code. The National Institutes of Health Collaboratory recommends assessing and reporting the quality of EHR data for clinical reuse in terms of data completeness, accuracy, and consistency [18]. Weiskopf et al [19] also determined that the completeness and correctness of data are of special importance for data quality improvement.

### Objective

To the best of our knowledge, there is no existing approach to systematically analyze and improve the structuredness of drug prescription data for observational research. Thus, in this study, we systematically analyzed the structuredness of EHR drug prescriptions to determine the ratio between structured drug prescription data containing ATC code level 5 and free-text drug prescriptions without an available standard concept based on the 14 ATC groups of level 1. In addition, we presented an approach to improve the structuredness of drug prescription data by introducing an automatic detection method for ATC code determination. To ensure the robustness and accuracy of the results of automatic detection, we introduced a validation step based on existing text-mining algorithms.

## Methods

### Study Details

This retrospective, noninterventional study systematically reviewed drug prescriptions based on real-world observational data at the University Hospital Carl Gustav Carus Dresden (UKD), Germany. This study was based on fully anonymized data and did not include any correlations with individual patients. All inpatient drug prescriptions, including acute medications, from 2016 to 2020 were included in the study, without restriction to specific conditions or treatments. The original data were recorded in the ORBIS hospital information system from Dedalus, using the ORBIS module, “KURV,” that represents the patient curve including medication data. A total of 1,768,153 drug prescriptions were reviewed from the hospital information system records. Drug prescription data from other systems (eg, intensive care units and chemotherapy) were excluded because data in those systems were completely structured and stored in separate backend systems. The data used for this study were provided by the Data Integration Centre at the UKD, which was established with funding from the German Federal Ministry of Education and Research as part of the Medical Informatics Initiative in Germany.

### Ethics Approval

The study was approved by the Ethics Committee of the Technical University of Dresden as a retrospective, observational, noninterventional, nonhuman subject study (SR-EK-521112021).

### Data Set Details

The following 2 data sets were used: drug prescription data (data set 1) and drug product catalog data (data set 2). The drug product catalog was exported from the UKD Enterprise-Resource-Planning system on November 16, 2021, and contained the drug product name, drug ingredient name, ATC level 5 code, drug dose and unit information, and legacy products. In addition, 2 other data sets were derived from data set 1. First, an aggregated data set (data set 3) was generated based on the grouped data set 1 for all the unstructured drug prescription entries. The grouping activity to create data set 3 was performed on the MEDICATION column of data set 1 by grouping all entries in the data element MEDICATION using the Python library Pandas and its *groupby* function. Frequency information for each unique MEDICATION record was added to data set 3. The data set (data set 4) contained a subset of the first 1000 most frequent entries from data set 3 and additional results from the manual evaluation step.

All the metadata elements of the data sets presented above that are relevant to this study are illustrated and described in detail in Table 1. Drug prescriptions selected from the drug catalog data are labeled as *structured data* (eg, “IBUPROFEN STADA 600 mg Zäpfchen | [Ibuprofen natrium, Ibuprofen]”) based on the contents of the STRUCTURE column of data set 1. Drug prescriptions that were not selected from the drug product catalog are designated as *unstructured data* (eg, “Ibuprofen 600” and “Ibuprofen”).

**Table 1:** Description of relevant data set with its metadata elements.

Data set and data element		Data type	Description
**DS^a^1 initial data set with all drug prescriptions**			
	MEDICATION	String	Free text or predefined value, chosen from an available fixed drop-down menu that contains product names, derived from the drug product catalog, when creating a new drug prescription
	YEAR	Number	Extracted from the prescription start date information for further statistical analyses
	STRUCTURE	Boolean	TRUE if MEDICATION was chosen from the drug catalog or FALSE if the free text was entered
	ATC^b^_L5	String	ATC code level 5 available in case STRUCTURE is true otherwise empty
**DS2 drug catalog data**			
	Product_name	String	Product name as listed in the ERP^c^ system
	Ingredient_name	String	Ingredient name as listed for the product
	Atc_code	String	ATC code level 5
**DS3 grouped DS1 by MEDICATION data element**			
	MEDICATION	String	Grouped unstructured free-text entries
	FREQUENCY	Number	Summed up the occurrence of the MEDICATION text field to determine the most relevant free-text drug prescriptions
	Step1	String	Algorithm 1 result as an ATC code or empty if no match
	Step2	String	Algorithm 2 result as an ATC code or empty if no match
	Step3	String	Algorithm 3 result as an ATC code or empty if no match
**DS4 most frequent 1000 entries of DS3 (sorted by frequency)**			
	MEDICATION	String	Grouped unstructured free-text entries
	FREQUENCY	Number	Summed up the occurrence of the medication text field
	Step1	String	Algorithm 1 result as an ATC code or empty if no match
	Step2	String	Algorithm 2 result as an ATC code or empty if no match
	Step3	String	Algorithm 3 result as an ATC code
	Eval1	Boolean	Algorithm 1 evaluation result
	Eval2	Boolean	Algorithm 2 evaluation result
	Eval3	Boolean	Algorithm 3 evaluation result
	True12	Boolean	TRUE if the same result for algorithm 1+2
	True13	Boolean	TRUE if the same result for algorithm 1+3
	True23	Boolean	TRUE if the same result for algorithm 2+3
	True123	Boolean	TRUE if the same result for algorithm 1+2+3
	CORRECT	String	Corrected ATC code. in case no algorithm determined the correct result, entered manually in the evaluation step
	COMMENTS	String	Any comments or additional information if needed
	FINAL	String	Finally determined ATC code for all entries or labels in case no ATC code could be determined (labels are introduced in the methods validation section in detail)

^a^DS: data set.

^b^ATC: Anatomical Therapeutic Chemical.

^c^ERP: Enterprise-Resource-Planning.

### Data Analysis

#### Overview

**Figure 1:**
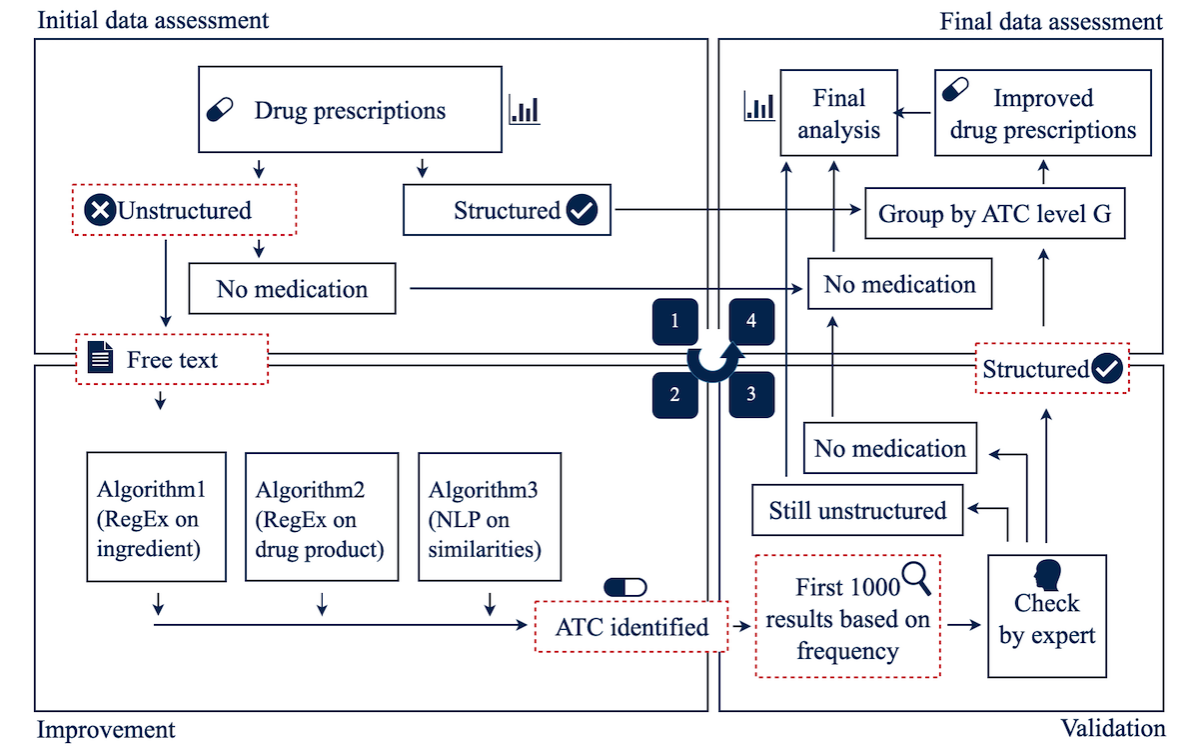
Data analysis 4-step approach. ATC: Anatomical Therapeutic Chemical; NLP: natural language processing.

Data analysis consisted of a 4-step process, as shown in Figure 1. The first step of the process was an initial data quality analysis to determine the overall ratio of structured to unstructured drug prescriptions.

To improve the structure of the drug prescriptions, 3 existing algorithms were applied to automatically identify correct ATC codes for the unstructured drug prescriptions. The identified ATC codes were then manually reviewed by experts (pharmacists and medical information scientists) and checked for correctness. This step also included the identification of existing patterns that can help conclude the reliability of the automatically identified ATC codes for unstructured data. Finally, the results of the previous 3 steps were consolidated to assess the degree of improvement achieved in unstructured drug prescriptions. To ensure expert coverage of the entire process, an interdisciplinary team of pharmacists, computer scientists, and medical informatics researchers was formed.

#### Initial Data Assessment

Initially, the ratio of structured to unstructured drug prescriptions was determined for data set 1. For this purpose, the STRUCTURE data element of data set 1 was used to subdivide the data into 2 groups. If the value of STRUCTURE was TRUE, the record was considered to be structured; otherwise, it was unstructured. Subsequently, the unstructured subset of drug prescriptions was grouped by the data element MEDICATION as data set 3, and the frequency was calculated and added as the data element FREQUENCY.

The first manual review of the grouped drug prescriptions (data set 3) was done by the interdisciplinary team of experts to identify records that are not drug prescriptions but other instructions, such as orders for blood counts or other laboratory and measurement orders (eg, “BGA”—laboratory request for blood gas analysis, “BE”—request to nurses for taking a blood sample, and “BB”—laboratory request for blood count). This task resulted in a set of rules (Multimedia Appendix 1) to allow the automated search and identification of medication entries that needed to be excluded for further steps.

#### Improvement

Unstructured drug prescriptions (usually provided as free text) were used as inputs for the improvement step. Preprocessing of the drug prescription was not performed previously. In this step, 3 different algorithms were implemented to automatically identify ATC level 5 codes based on the MEDICATION text.

The algorithms were based on a different mechanism for matching the MEDICATION text of data set 1 with the product catalog data elements INGREDIENT_NAME and PRODUCT_NAME of data set 2, as described in detail in Table 2.

**Table 2:** An overview of algorithms for Anatomical Therapeutic Chemical (ATC) code identification for unstructured drug prescriptions.

Algorithm	Mechanism	Data input for comparison		Result data
		Data set 1	Data set 2	
1	String comparison	MEDICATION	Ingredient_name	ATC code
2	String comparison	MEDICATION	Product_name	ATC code
3	Similarity matching	MEDICATION	INGREDIENT_NAME and PRODUCT_NAME	ATC code+similarity score

Algorithms 1 and 2 rely on simple string comparisons to recognize either the ingredient name or product name within the drug prescription. Algorithm 3 performs natural language processing (NLP) based on similarity matching between the data element MEDICATION in data set 1 and the 2 data elements PRODUCT_NAME and INGREDIENT in data set 2 with the Python library *FuzzyWuzzy* [20] using Levenshtein distance because it has shown promising results in other health care research areas [21,22]. The best similarity score result was 100, which meant that the components of the string MEDICATION were entirely contained in INGREDIENT_NAME or PRODUCT_NAME. The lower the similarity score, the less similar the MEDICATION string is compared with the drug catalog entries. This algorithm provided up to 3 possible ATC codes, sorted in descending order based on their similarity scores. To determine the most promising method of the *FuzzyWuzzy* library for our implementation, we defined that the word order in the data element MEDICATION is irrelevant and can be different from the compared strings in INGREDIENT_NAME and PRODUCT_NAME. All words from the entry of the data element MEDICATION must be included in the entry of INGREDIENT_NAM*E* or PRODUCT_NAME, but not vice versa. This led to the implementation of the method *token_set_ratio*. This method tokenizes both strings to be compared, changes the upper case to the lower case, and removes punctuation. It then sorts the tokens alphabetically and split them into 2 groups: the intersection group (tokens that are the same in both strings) and the remainder group (tokens that differ in compared strings). The *token_set_ratio* method compares the intersection group with the intersection and remainder of the first string and then the same with the remainder of the other string and finally takes the highest results of this comparison as the final result. As shown in the following example (Textbox 1), the *token_set_ratio* method provides the best results concerning the given requirements.

**An example of the token_set_ratio method.**

d1 = “Stada paracetamol”

d2 = “paracetamol Stada 400 mg”

Print(“Ratio: ”, fuzz.ratio(d1.lower(),d2.lower()))

Print(“Partial Ratio: ”, fuzz.partial_ratio(d1.lower(),d2.lower()))

Print(“Token Sort Ratio: ”, fuzz.token_sort_ratio(d1.lower(),d2.lower()))

Print(“Token Set Ratio: ”, fuzz.token_set_ratio(d1.lower(),d2.lower()))

Ratio: 54

Partial Ratio: 65

Token Sort Ratio: 83

Token Set Ratio: 100

The algorithms were applied to data sets 1 and 3. The results of the algorithms in data set 3 were also used in data set 4. The concordance between the results for each permutation (algorithms 1+2, 1+3, 2+3, and 1+2+3) was also calculated. The complete source can be accessed on Zenodo [23].

#### Validation

The validation step consisted of manual checks of the automatically generated ATC codes by the same interdisciplinary team as in the previous steps. It was performed on a subset of the most common free-text prescriptions. To maintain the validation effort proportionate to the benefit, a minimum target was defined for the manual validation process of unstructured drug prescriptions to cover at least 80% of structured and manually validated unstructured entries combined. During the validation step, information was added to each algorithm to determine whether the correct ATC code, wrong ATC code, or no ATC code was identified. If no algorithm identified the correct ATC code, it was determined by manual validation when possible. If an entry was found to generally have no drug prescription, it was marked as an additional entry without drug prescription with the keyword “nomed.” For drug prescriptions that require further specification to determine the exact ATC level 5 code, the manual review checks whether the ATC level 4 or 3 code can be determined based on the free text of the drug prescription, otherwise the entry was flagged as unspecific with the keyword “unspec.” All unstructured drug prescription entries for which no validation of the automatically generated ATC codes was performed were marked with the keyword “no_eval*.*”

The results of the manual validation were summarized to identify any patterns that can help improve the robustness of the results of automatically detected ATC codes based on the total findings and correctness of each algorithm, the concordance level between the results of algorithms 1, 2, and 3, and the Levenshtein similarity score for algorithm 3. For algorithm 3, we used a 2-tailed *t* test implemented in Python to determine whether there was a significant difference between the means of the Levenshtein similarity score for the correct and incorrect results.

In addition, the incorrect results were examined in more detail by the interdisciplinary team to identify patterns that would reveal important reasons and similarities related to the ingredients of concern (ATC) to the greatest extent possible.

#### Final Data Assessment

For the final data assessment, the results of the step improvement and validation documented in data set 4, including correctly identified ATC level 5 codes or “nomed,” “unspec,” or “no_eval” labels, were merged with the original drug prescription data from data set 1. Thus, the final data assessment was executed based on the algorithm results and manual validation. The total number of drug prescription records was determined for each of the 14 ATC groups, including the proportion of structured versus unstructured data per ATC group. In addition, the total number of unique ATC level 5 codes, including their structuredness, as well as the most frequent ATC level 5 codes used in drug prescription of the data set 1 are presented. This allows a ranking of the structuredness based on the ATC groups and ATC codes.

## Results

### Initial Data Assessment

The initial assessment revealed 843,980 (n=1,768,153, 47.73% drug prescriptions in the data set 1) structured drug prescriptions. The proportion of unstructured drug prescriptions that required further investigation was 52.27% (924,173 drug prescription entries). A small set of rules, for example, all drug prescription entries starting with laboratory or measurement orders (Multimedia Appendix 1), identified a total of 160,896 (9.1% of all drug prescriptions) entries as no drug prescription data and reduced the unstructured drug prescriptions requiring review for the next steps to 763,277 (43.17% of all drug prescriptions).

Grouping the unstructured drug prescriptions based on the MEDICATION data element of data set 1 resulted in a total of 100,004 (n=924,173, 10.82%) unique free-text entries that were entered as medication prescription information and stored as data set 3 after adding the frequency for each free text.

### Improvement

The quantitative performance of the algorithms was very different, as each algorithm returned a different number of results. Algorithm 3 provides an ATC code for all unstructured drug prescriptions, owing to its implementation and nature.

**Figure 2:**
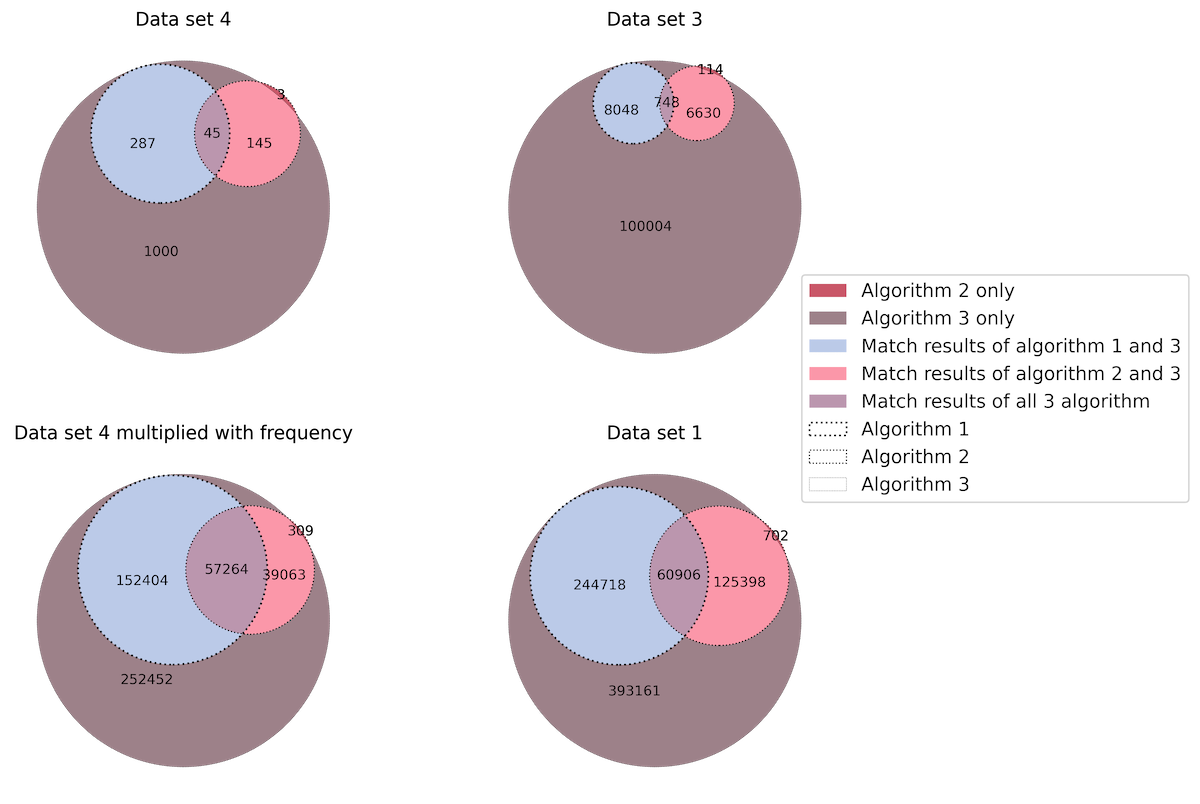
Match rates of algorithm results calculated for all data sets under inspection.

Algorithm 1 (based on ingredient matching) identified ATC codes for 8048 unique free texts. Multiplied by the frequency of each text entry, this yielded a total of 244,718 (32.06%) drug prescriptions of the total 763,277 unstructured drug prescriptions. The quantitative outcome performance of algorithm 2 (based on the drug product) is lower than that of algorithm 1, as it identified ATC codes for 6744 unique free texts. This represents a total of 126,100 (16.52%) drug prescriptions of the total 763,277 unstructured drug prescriptions. At this point, no statement can be made about the correctness of the algorithm results, but the analysis of the match rate between all algorithms shows matching rates for the total number of unstructured drug prescriptions and the most frequent 1000 free-text entries as illustrated in Figure 2.

### Validation

**Figure 3:**
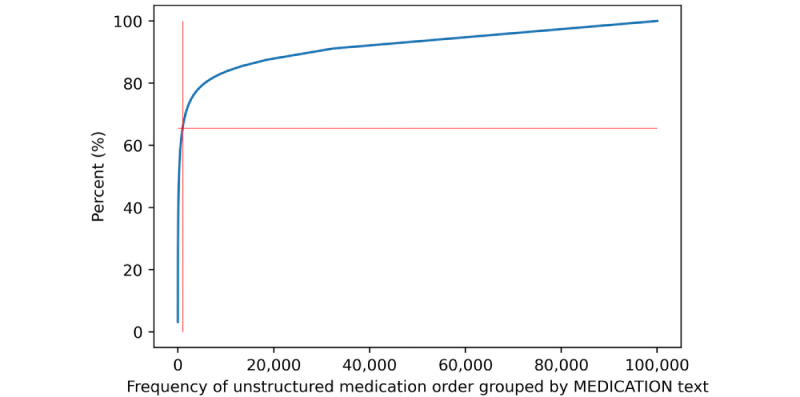
Percentage of the most frequent 1000 of all unstructured drug prescriptions.

The manual validation step was performed on the most frequent 1000 free-text entries, which already covered 66.56% (615,129/924,173) of all unstructured drug prescriptions, as shown in Figure 3. Together with the proportion of structured drug prescriptions (843,980/1,768,153, 47.73%) and entries without medication (166,307/1,768,153, 9.4%) that were identified during the initial data analysis, the structuredness could potentially be increased to 85.18% (1,506,059/1,768,153) of all medication prescriptions.

For the most frequent 1000 free-text entries (data set 4), algorithm 1 returned 286 (28.6%) correct results, 1 (0.1%) incorrect result, and no results for 713 (71.3%) entries. Algorithm 2 returned 142 (14.2%) correct results, 6 (0.6%) incorrect results, and no results for 852 (85.2%) unique entries. Algorithm 3 returned 765 (76.5%) correct results and 235 (23.5%) incorrect results. We also determined the correctness in terms of the result match rates between the algorithms, as shown in Figure 2, for data set 4. After the manual validation of data set 4, the returned ATC codes were always correct if all algorithms or algorithms 1 and 2 returned the same results.

For the matching results of algorithms 1 and 3, we noted a minor discrepancy and identified 5 incorrect results out of 286, which were related to sodium chloride drug prescriptions in 4 out of 5 cases, and another incorrect result was returned for the ingredient aciclovir. The manual review revealed that an ATC code could not be provided because of missing details that was due to the ATC code at ATC level 1 varying by route of administration (eg, oral, parenteral, and conjunctival). For the matching results of algorithms 2 and 3, we identified only 1 incorrect result related to the ingredient telmisartan because the drug prescription was for a combination drug (telmisartan and diuretics), whereas the algorithms identified only the ATC code of the single ingredient telmisartan.

For the data set 4, a significant difference in the means of the Levenshtein similarity score was found between the correct and incorrect results (see Table 3 for descriptive statistics) with a *P* value of 2.4 × 10^–47^, which is well below the significance level α (.05). This means that the higher the Levenshtein similarity score, the higher the probability of result correctness. Speaking in terms of absolute numbers, for entries with a Levenshtein similarity score >84.28, the results can be assumed to be correct with a low error rate.

**Table 3:** Descriptive statistics for Levenshtein similarity score for correct and wrong results.

Descriptive statistics			Algorithm 3		
			Correct		Wrong
Count			766		234
Frequency, n (%)			416,585		84,598
Values, mean (SD)			84.28 (14.86)		67.18 (15.52)
Range (%)			21-100		29-100
**Percentile**					
	25th percentile	76		55	
	50th percentile	87		63	
	75th percentile	96		75	

The 234 incorrect results returned only by algorithm 3, with 4.78% (84,598/1,768,153) of drug prescriptions, can be categorized into four groups: (1) manually identified additional entries without drug prescriptions for which the applied rules did not work; (2) specification generally not possible owing to missing information; (3) restriction to ATC level 3 or 4 because of nonspecific drug prescription information; and (4) other reasons. We found 16 entries (multiplied by frequency=5411) with no additional drug prescription entries. For an additional 11 (multiplied by frequency=2187) entries, no ATC code could be provided because the dosage form or dose was missing. For 2 drug prescriptions (multiplied by frequency=2610) of insulin therapies, there was a restriction to ATC level 3. Another 2 (multiplied by frequency=887) entries were restricted to ATC level 4 sodium chloride prescriptions.

For the other 203 misidentified entries, we examined the subset of 26 entries where algorithm 3 returned results with a Levenshtein similarity value of ≥80 because it is an indication of correctness but unfortunately did not apply to all results. A small group of 26 results with a Levenshtein similarity value of ≥80 was still incorrect. The main reason for the errors in these results was that the ATC codes for the ingredients differed by dosage form and when combined in a drug product, as shown in Table 4. Most incorrect results (15 out of 26) were caused by the absence of the ingredient dosage form in the free text, especially for sodium chloride, prednisolone, dimetindene, aciclovir, and hydrocortisone. The full data set 4 with all the algorithm outcome quality data elements listed in Table 1 is available in Multimedia Appendix 2.

**Table 4:** Wrong results of algorithm 3 with Levenshtein similarity score ≥80.

Medication free text	Wrong result	Levenshtein similarity score	Correct result	Reason
ASS RATIOPHARM 100 mg TAH Tabletten|(Acetylsalicylsäure)	N02BA01	89	B01AC06	Similarity of words
Prednisolon	S01CA53	100	H02AB06	Dosage form
MAGNESIUM VERLA 300 Orange Granulat|(Magnesium-Ion)	A12CC05	100	V06XX02	Similarity of words
ARILIN 500 Filmtabletten | (Metronidazol)	G01AF01	100	P01AB01	Similarity of words
CANDESARTAN HEXAL comp 16 mg/12.5 mg Tabletten | (Candesartan)	C09CA06	89	C09DA26	Combination product
Heparin	C05BA03	100	B01AB01	Dosage form
PREDNISOLON	S01CA53	100	H02AB06	Dosage form
FENISTIL Injektionslösung | (Dimetinden)	D04AA13	100	R06AB03	Dosage form
ACIC 250 PI Via Pulver z.Herst.e.Infusionslösg. | (Aciclovir)	D06BB03	100	J05AB01	Dosage form
NaCl 0.9%	B05CB01	100	B05BB11	Dosage form
VALSARTAN HEXAL comp.160mg/12,5mg Filmtabletten | (valsartan)	C09CA03		C09DA23	Combination product
Prednisolon mg	S01CA53	88	H02AB06	Dosage form
NaCL 0.9%	B05CB01	100	B05BB11	Dosage form
ACIC 200 Tabletten | (Aciclovir)	D06BB03	100	J05AB01	Dosage form
ACIC 500 PI Via Pulver z.Herst.e.Infusionslösg. | (Aciclovir)	D06BB03	100	J05AB01	Dosage form
Simvastatin	C10BA02	100	C10AA01	No combination product
CANDESARTAN HEXAL comp 8 mg/12.5 mg Tabletten | (Candesartan)	C09CA06	89	C09DA26	Combination product
NaCL 0.9% | (Natrium-Ion, Chlorid)	B05CB01	100	B05BB11	Dosage form
C) FENISTIL 1 Ampulle als Bolus | (Dimetinden)	D04AA13	100	R06AB03	Dosage form
HCT	C09DX01	100	C03AA03	Shortness of text
Allopurinol	M04AA51	100	M04AA01	Combination product
Prednisolon 5 mg	S01CA53	81	H02AB06	Dosage form
HYDROCORTISON 10 mg Jenapharm Tabletten | (Hydrocortison)	S01BA02	81	H02AB09	Dosage form
Simvastatin 20 mg	C10BA02	100	C10AA01	No combination
NaCl 0.9%	B05CB01	100	B05BB11	Dosage form

### Final Data Assessment

Compared with the initial data assessment in which we could only distinguish between structured and unstructured drug prescriptions, we were able to perform a percentage distribution between structured and unstructured drug prescriptions for each of the 14 ATC level 1 groups after applying the algorithm. The final results are presented in Table 5, which shows the number of structured drug prescriptions versus the number of unstructured drug prescriptions per ATC level 1 group. The total number of drug prescriptions per ATC level 1 group, including percentages, provides an overview of the most and least frequently prescribed drugs sorted by the 14 ATC level 1 groups. For completeness, we added 3 additional rows to Table 5 containing the number of unstructured entries identified as other orders (no_med), the number of unstructured entries identified as unspecified entries (unspec), and the remaining unstructured data for which no validation was performed; thus, no statement on the correct ATC code was possible. ATC level 1 group “N – Nervous system” was the most common group with 24.1% (322,286/1,337,565) of the initial data set 1, followed by “B – Blood and blood forming organs,” “A – Alimentary tract and metabolism,” and “C – Cardiovascular system” with approximately 19% each.

**Figure 4:**
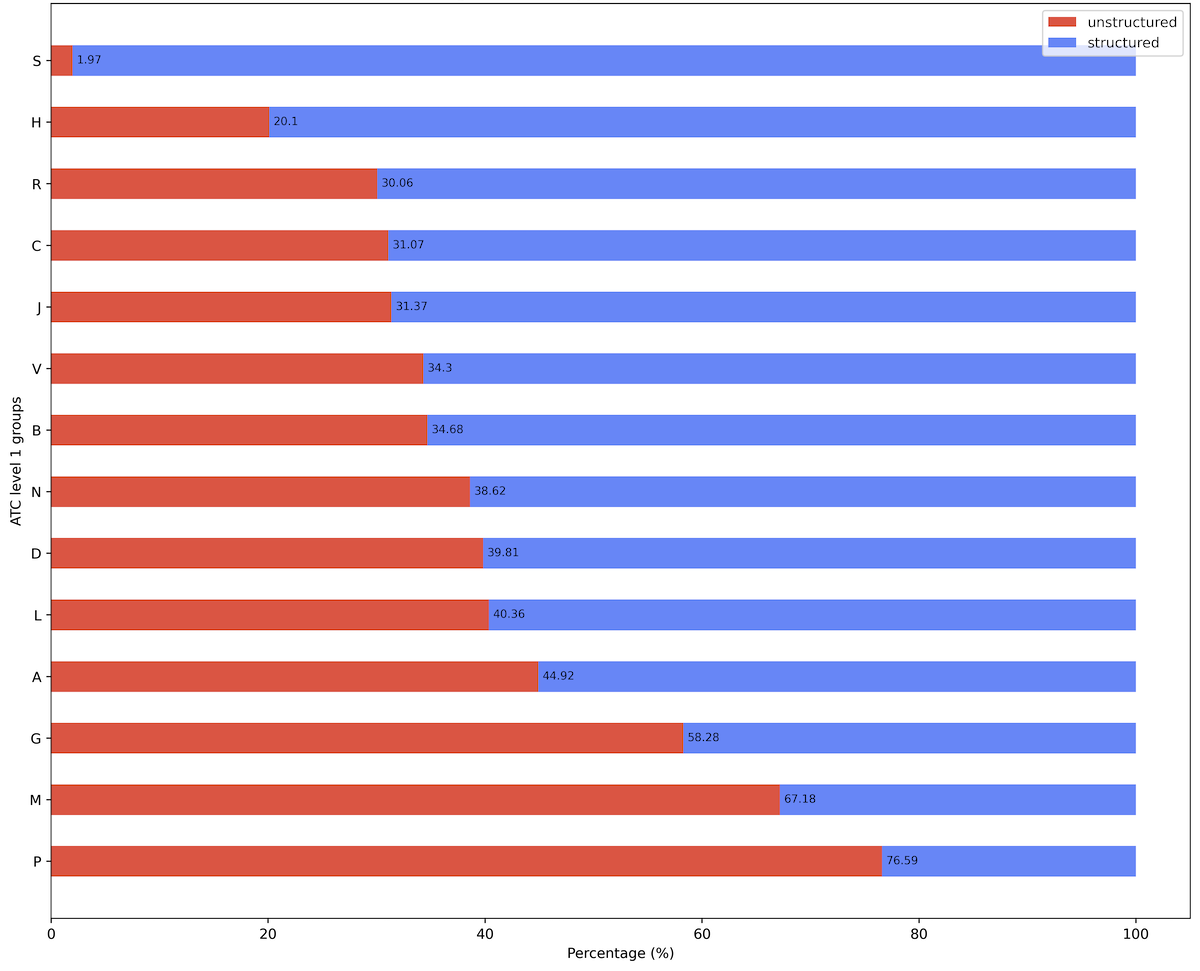
Structuredness of drug prescriptions by ATC groups for 85.18% of initial data set DS1. ATC: Anatomical Therapeutic Chemical.

Figure 4 illustrates the structuredness of the data for each of the 14 ATC level 1 groups. The ATC level 1 group with the most structured data was “S – Sensory organs” with 98.03% (5077/5179) structured data, followed by the group “H – Systemic hormonal drugs, excluding sex hormones and insulins” with 79.9% (51,296/64,199) structured data. ATC level 1 groups “R – Respiratory system,” “C – Cardiovascular system,” “J – Anti-infective for systemic use,” “V – Various,” ”B – Blood and blood forming organs,” and “N – Nervous system,” ranged from 61% to 70% structured data. ATC group “P – Antiparasitic products, insecticides, and repellents” had the lowest percentage of structured drug prescriptions at only 23.4% (342/1461).

In total, 742 ATC level 5 codes (ingredients) were identified in the drug prescription data.

**Figure 5:**
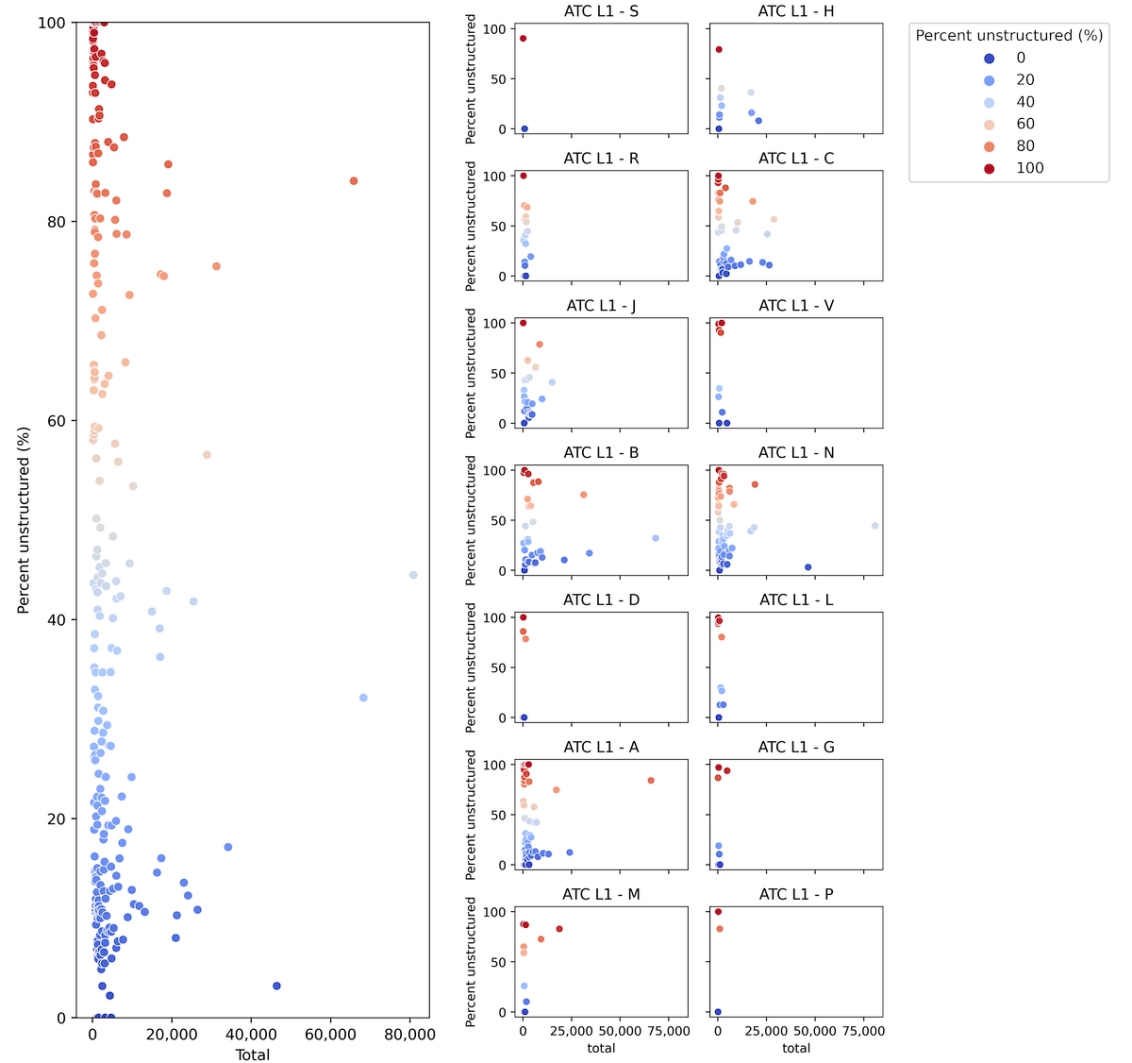
Structuredness of drug prescription by ATC L5 (a) total unstructured data and (b) by each ATC L1 group. A: Alimentary tract and metabolism; ATC: Anatomical Therapeutic Chemical; B: Blood and blood forming organs; C: Cardiovascular system; D: Dermatologicals; G: Genito urinary system and sex hormones; H: Systemic hormonal drugs, excluding sex hormones and insulins; J: Anti-infective for systemic use; L: Antineoplastic and immunomodulating agents; L1: level 1; L5: level 5; M: Musculo-skeletal system; N: Nervous system; P: Antiparasitic products, insecticides, and repellents; R: Respiratory system; S: Sensory organs; V: Various.

The structuredness of the ingredients varied widely, showing a wide range of structuredness among ingredients, as shown in Figure 5, where each of the 742 ATC level 5 codes (ingredients) is represented by a single dot. The y-axis represents the degree of the structuredness between 0% and 100%. The x-axis represents the frequency of each ATC level 5 code in data set 1, with a limitation of 85.18% (1,506,059/1,768,153) structured and evaluated unstructured data. There were only 4 ATC level 5 codes that were each used more than 45,000 times in drug prescriptions, namely N02BB02 (metamizole), B05BB01 (sodium chloride), A02BC02 (pantoprazole), and N02AA05 (oxycodone).

**Table 5:** Number of Anatomical Therapeutic Chemical (ATC) codes by ATC level 1 for structured, unstructured, and combined data.

ATC 1st level	Structured drug prescriptions (n=843,980), n/N (%)	Unstructured drug prescriptions for evaluated subset (n=924,173), n/N (%)	Total number (n=1,768,153), n/N (%)
N – Nervous system	197,831/322,286 (61.38)	124,455/322,286 (38.62)	322,286/1,337,565 (24.1)
B – Blood and blood forming organs	164,032/251,120 (65.32)	87,088/251,120 (34.68)	251,120/1,337,565 (18.77)
A – Alimentary tract and metabolism	137,988/250,543 (55.08)	112,555/250,543 (44.92)	250,543/1,337,565 (18.73)
C – Cardiovascular system	170,703/247,629 (68.93)	76,926/247,629 (31.07)	247,629/1,337,565 (18.51)
J – Anti-infective for systemic use	60,844/88,659 (68.63)	27,815/88,659 (31.37)	88,659/1,337,565 (6.63)
H – Systemic hormonal drugs, excluding sex hormones and insulins	51,296/64,199 (79.9)	12,903/64,199 (20.1)	64,199/1,337,565 (4.8)
M – Musculo-skeletal system	12,083/36,819 (32.82)	24,736/36,819 (67.18)	36,819/1,337,565 (2.75)
R – Respiratory system	19,686/28,148 (69.94)	8462/28,148 (30.06)	28,148/1,337,565 (2.1)
V – Various	9639/14,672 (65.7)	5033/14,672 (34.3)	14,672/1,337,565 (1.1)
L – Antineoplastic and immunomodulating agents	8670/14,538 (59.64)	5868/14,538 (40.36)	14,538/1,337,565 (1.09)
G – Genito urinary system and sex hormones	3662/8778 (41.71)	5116/8778 (58.28)	8778/1,337,565 (0.66)
S – Sensory organs	5077/5179 (98.03)	102/5179 (1.97)	5179/1,337,565 (0.39)
D – Dermatologicals	2127/3534 (60.19)	1407/3534 (39.81)	3534/1,337,565 (0.26)
P – Antiparasitic products, insecticides, and repellents	342/1461 (23.41)	1119/1461 (76.59)	1461/1,337,565 (0.11)
no med	0/1,768,153 (0)	166,307/1,768,153 (9.41)	166,307/1,768,153 (9.41)
unspec	0/1,768,153 (0)	2187/1,768,153 (0.12)	2187/1,768,153 (0.12)
Total validated	843,980/1,768,153 (47.73)	662,079/1,768,153 (37.44)	1,506,059/1,768,153 (85.18)
Not validated	0/1,768,153 (0)	262,094/1,768,153 (14.82)	262,094/1,768,153 (14.82)

Together, these 4 ATC level 5 codes accounted for 14.79% (261,460/1,768,153) of the total data in the data set 1. Pantoprazole had the lowest level of structured data for these 4 ingredients (10,490/65,861, 15.93%), whereas oxycodone had the highest level of structured data (44,952/46,434, 96.81 %). Multimedia Appendix 3 contains the complete list of all ingredients with their ATC level 5 codes, number of structured, unstructured, and total drug prescriptions. In addition, it includes the percentage strength of each ingredient, ordered by the total number of drug prescriptions, starting with the largest number.

## Discussion

### Principal Findings

Our 4-step approach ensures data quality assessment as recommended by Zozus et al [18]. We provide transparency by reporting the structuredness of drug prescriptions. In addition, our approach improved the structuredness and thus the completeness of drug prescription data. This leads to better usability for secondary use on research infrastructure, such as the OMOP based ATC codes accompanied by manual review.

The initial analysis of the drug data showed a ratio of 52.3% unstructured to 47.7% structured drug prescriptions. With the algorithms presented, the structuredness could be increased to 85.1% with 1 level of evaluation. For the evaluation of the initial data set 1, manual examination of the most frequent 1000 free-text entries was sufficient, and we were able to achieve the targeted minimum coverage of 80%. Algorithm 3, which was based on similarity matching, was found to quantitatively outperform the other 2 algorithms, providing results for all unstructured drug prescriptions. In terms of the reliability of the results, algorithm 3 had a correctness rate of only 76.5%. Therefore, the evaluation phase was critical to manually correct all incorrectly derived ATC codes. In addition, the manual evaluation process was critical in identifying patterns that can be used to determine the reliability of the algorithms based on additional factors, such as algorithm-to-algorithm matches and the Levenshtein similarity score of algorithm 3. When all 3 methods or algorithms 1 and 2 yielded the same ATC code, the algorithm results were considered correct in each case. The scoring process yielded a minuscule percentage of incorrect results (approximately 1.5%) when algorithms 2 and 3 or 1 and 3 yielded the same results.

The patterns identified are a good indication that can help increase the reliability of the results without further manual evaluation, compared with the overall correctness of 76.5% (765/1000) found for algorithm 3 results. The Levenshtein similarity score revealed another trend for algorithm 3: incorrect results had a significantly lower mean similarity score than correct results. The exceptions have been isolated to a small number of ingredients, reasons such as missing dosage form and dosage information, and cases where the same ingredients were used for single and combination drug products, resulting in separate ATC codes. The quality of the RWD has a major impact on the results of observational studies that rely on it. It is important to ensure that RWD data are suitable for use in observational studies [24] and that any limitations or quality concerns are explicitly stated [25].

### Limitations

Currently, the analyzed data set is limited to inpatient drug prescriptions from the University Hospital Carl Gustav Carus Dresden. No drug prescription data from intensive care medicine were included, and no other institution has used our technique yet. Outliers or rare patterns may have gone undetected because the study was limited to the first 1000 free-text prescriptions. Although this covers most of the data, the results of the algorithm for the remaining free-text entries are yet to be evaluated. This study does not include an outcome evaluation of additional drug prescription entries based on identified patterns. Currently, the method is limited to determining ATC codes for unstructured drug prescriptions and does not consider other terminologies such as RxNorm.

### Comparison With Prior Work

Most studies evaluating the quality of RWD data refer to the dimensions of completeness and accuracy compared with predefined gold standard data that vary by publication, as identified by Weiskopf et al [19]. We did not define our gold standard based on RWD sources but used the internationally recognized and widely used terminology ATC as standardized terminology and provided a method to automatically determine the appropriate ATC for drug prescription data that are unstructured and available only as free text. Wang et al [26] developed a rule-based data quality system with >6000 criteria for plausibility testing (eg, pregnancy is not plausible in male patients) but did not address data harmonization by mapping unstructured free-text data to defined terminologies for research. Unlike Schmidt et al [27] and Kahn et al [25], our study not only focus on data quality assessment but also defines the absence of structure in the data as free text without a corresponding ATC code and builds on previous research by proposing a method to improve unstructured data by automatically annotating the appropriate ATC code.

The high proportion of free-text or unstructured drug prescriptions was due to the hospital’s prescription system and local conditions. According to previous studies on the data structure in RWD [28], this is a widespread challenge in Germany. However, the issues of dealing with unstructured data in EHR records that prevent interoperability are widespread as stated by Kruse et al [29,30] in their systematic reviews of existing literature on the use of EHR data that need to be addressed to ensure “fitness for use” in general. Compared with the well-established Unified Medical Language System MetaMap [31,32], which has been used by industry and academia for many years, our NLP approach focuses on a lightweight implementation. On the one hand, this limits the configuration possibilities, but on the other hand, it reduces the computational efforts and promotes the performance of the ATC code recognition. Because MetaMap focuses only on English and does not support German drug catalogs, our approach closes this gap and can be adapted to other languages as well.

### Future Work

The presented 4-step approach can be applied to any RWD with unstructured data such as conditions, procedures, or test results. This approach will be tested in the future on other sites that provide drug data and product lists with ATC codes. In the next phases, further research will be conducted on pattern recognition to enable reliable prediction of the accuracy of results for specific ATC codes rather than manually checking them. In addition, new NLP-based algorithms will be implemented to improve the overall reliability of the results. Furthermore, our approach can be applied to other hospital sites that participate in the German Medical Informatics Initiative [33,34] in the following steps. Our approach is not limited to the German language. Because the only requirement is to provide a common list of ingredients or drug products for comparison with unstructured free text, this can work for any other language if compared texts are available in the same language.

### Conclusions

RWD observational research requires a high level of data structuredness. Even more critical is the awareness of limitations as well as transparency of the level of structure of the data on which the research is based. Using drug prescriptions as a first use case, we were able to investigate and improve the structure of RWD, which can be applied to other RWDs in the future. Although the presented methods require manual verification to ensure that the results are correct, the methodology is promising and can be used to improve structuredness of data.

## Appendix

Multimedia Appendix 1 Small set of rules.

Multimedia Appendix 2 Data set 4.

Multimedia Appendix 3 Complete list of ATC L 5 with frequency and proportion of structured versus unstructured entries.

## Abbreviations

ATC: Anatomical Therapeutic Chemical
EHR: electronical health record
NLP: natural language processing
OMOP: Observational Medical Outcomes Partnership
RCT: randomized controlled trial
RWD: real-world data
UKD: University Hospital Carl Gustav Carus Dresden
